# miR-937 serves as an inflammatory inhibitor in cigarette smoke extract-induced human bronchial epithelial cells by targeting IL1B and regulating TNF-α/IL-17 signaling pathway

**DOI:** 10.18332/tid/138227

**Published:** 2021-06-25

**Authors:** Teng Liu

**Affiliations:** 1 Department of Respiratory Medicine, Shandong Provincial Chest Hospital, Shandong University, Jinan, China

**Keywords:** hsa-miR-937, chronic obstructive pulmonary disease, Interleukin 1β, proliferation, apoptosis

## Abstract

**INTRODUCTION:**

This study aimed to elucidate the biological implication of miR-937 in cigarette smoke extract (CSE)-induced human bronchial epithelial (HBE) cells and to further investigate its possible regulatory mechanism.

**METHODS:**

Public datasets were downloaded to identify differentially expressed genes and subjected to Kyoto encyclopedia of genes and genomes (KEGG) pathway enrichment analysis in chronic obstructive pulmonary disease (COPD). Online prediction site and luciferase reporter assay were applied to determine the target correlation between miR-937 and IL1B. RT-qPCR, Western blot and Enzyme-Linked Immunosorbent Assays (ELISA) analyses were used to evaluate the expressions of indicated molecules. HBE cells were exposed with CSE (20 μg/mL) to construct the in vitro COPD model. Cell proliferation and apoptosis were measured through cell counting kit 8 and Annexin-V/propidium iodide (PI) staining assays.

**RESULTS:**

IL1B was found to be up-regulated in COPD samples compared with healthy controls and had a high correlation with the TNF and IL-17 pathways according to the data from GSE57148. Moreover, IL1B was predicted to be a target of miR-937, and it was negatively regulated by miR-937. CSE treatment reduced the miR-937 expression, meanwhile decreased the HBE cells proliferation, enhanced cells apoptosis, and elevated the expression of IL-6, IL-17, and TNF-α. Moreover, in the CSE model, upregulation of miR-937 promoted cells viability, restrained cells apoptosis, and decreased levels of IL-6, IL-17, and TNF-α were noted, which could be abolished by overexpression IL1B. In contrast, inhibiting miR-937 impeded cells proliferation, promoted cells apoptosis and elevated levels of IL-6, IL-17 and TNF-α, which could be rescued by IL1B-knockdown in CSE-induced HBEs.

**CONCLUSIONS:**

These findings suggest that miR-937 plays a protective role on the HBEs after CSE damage, which may be achieved via targeting IL1B and inhibiting the TNF-α/IL-17 signaling pathway.

## INTRODUCTION

Chronic obstructive pulmonary disease (COPD) has been recognized as a common respiratory disease characterized by progressive, incomplete reversible airflow limitation^1^. It is frequently associated with chronic inflammation, emphysema, and loss of lung function^2^. Among them, chronic inflammatory injury is still the core-pathogenesis of COPD progression that can be further developed into chronic pulmonary heart disease and respiratory failure^3^. Alarmingly, the morbidity and mortality of COPD are increasing year by year^4^. In China, COPD accounts for nearly 1 million deaths annually and represents 30% of all deaths from COPD worldwide^5^. Numerous factors contribute to COPD, including cigarette smoke, secondhand smoke, occupational exposure, exhausts and fuel, age, and genetics, among which cigarette smoke is the important contributor for its development^6,7^. However, the underlying mechanisms for the development of COPD remain largely unknown.

Interleukin 1β (IL1B), known as a proinflammatory cytokine, is of importance to both normal immune responses and chronic inflammatory diseases^8^. It is synthesized by a variety of cell types, including blood monocytes and tissue macrophages^9^. Importantly, multiple reports have verified that IL1B is an important mediator in cigarette smoke (CS)-induced inflammation and COPD and plays a crucial role in initiating and maintaining airway inflammation^10,11^. In CS-exposed mice, the level of IL1B was significantly increased compared with sham group^12^. Besides, IL1B was identified to be a biomarker of bacteria-, virus- or eosinophil-associated exacerbations of COPD^13^. In COPD, IL1B level, exceeding its antagonists, was related to disease severity, including BMI and FEV1^14^. IL1B is a highly inflammatory cytokine, any change in its concentration in the blood or tissue may have a dramatic impact on the pathogenesis of COPD. However, the molecular mechanism mediating the abnormal inflammatory responses in COPD is still elusive.

MicroRNAs (miRNAs), endogenous small non-coding RNAs of less than 22 nucleotides in length, are well known to act as important factors in multiple diseases. Generally, they play their roles by binding to the 3'UTR structure of the target mRNA, thereby leading to mRNA degradation or restraining post-transcriptional translation. Accumulating evidence has supported that a crowd of miRNAs are of importance to the development of COPD, revealing promise for their future application in COPD treatment. Additionally, it has been shown that miRNAs participate in the modulation of the inflammatory immune response, which is a characteristic of COPD^15^. For instance, miR-132 was found to be highly expressed in COPD patients and smokers, in contrast with non-smokers, and promote inflammation in human monocyte-like cells and bronchial epithelial cells by targeting suppressor of cytokine signaling 5^16^. A study has demonstrated that miR-29b is involved in airway inflammation by mediating the expression of inflammatory cytokine via targeting bromodomain-containing protein 4 in COPD^17^. Noticeably, using the DESeq2 method, miR-937 was identified to be downregulated in COPD patients compared with non-smokers^18^. But the role and the exact mechanism of miR-937 affecting the development of COPD remain unclear.

Fortunately, with the help of online prediction tools, the data showed that IL1B was a target gene of miR-937, as IL1B is an important mediator in cigarette smoke extract (CSE)-induced inflammation and COPD, and downregulation of miR-937 might be involved in the development of COPD. Thus, we hypothesized that miR-937 might affect the progression of COPD and inflammation response via regulating the expression of IL1B. However, related evidence is limited. In this study, the role and the potential mechanism of miR-937/IL1B in CSE-damaged HBE cells were probed, which may be helpful for understanding the pathogenesis of COPD in the hope of developing novel pathways for COPD treatment.

## METHODS

### Data acquisition

Gene expression dataset GSE57148 and miRNA expression profile GSE38974 were derived from the Gene Expression Omnibus (GEO, https://www.ncbi.nlm.nih.gov/geo/) database. GSE57148 includes the mRNA expression profile from 9 samples from healthy lung tissue and 23 samples from COPD cases. GSE38974 comprises the noncoding RNA profile by array from 19 samples from COPD patients and 9 normal samples from lung tissue.

### Data preprocessing and analysis of differently expressed genes (DEGs)

The .txt files of DEGs for the two microarray datasets were acquired using the Limma package in R language^19^. The raw data were corrected for background noise and normalized using the Robust Multi-array Average (RMA) method. Significant DEGs were identified when the adjusted p-value was less than 0.05, and the absolute log FC ≥1. The cluster Profiler package was recruited to conduct Kyoto encyclopedia of genes and genomes (KEGG) pathway analysis for DEGs^20^.

### Preparation of CSE

CSE was prepared by a method from a previous study^21^. Briefly, a standard Double Happiness cigarette (Shanghai Tobacco Corporation, China, nicotine: 1.0 mg/cigarette) was attached to a smoking device and lit. CS was extracted through a filter for removing the bacteria and impurities into a vessel using a vacuum pump and dissolved in 37^o^C serum-free minimum Eagle’s medium (MEM, Gibco, Rockville, MD, USA). The obtained CSE solution was adjusted to a pH of 7.4 and used as the mother liquor at the dose of 1 mg/mL, and stored in a -80^o^C refrigerator in the dark.

### Cell growth condition and CSE exposure

Human bronchial epithelium (HBE) cells were obtained from ATCC (Rockville, IN, USA) and incubated in RPMI-1640 medium (Procell, Wuhan, China) containing 10% fetal bovine serum (FBS) and 1% penicillin/streptomycin solution. Cells were incubated in a 37°C incubator under 5% CO_2_ atmosphere. After incubation for 24 hours, cells were exposed with 20 μg/mL cigarette smoke extract (CSE) for 24 hours and harvested for the following experiments.

### Cell transfection

Cell transfection was carried out when the cells concentration reached 70–80%. Briefly, cells were transfected with miRNA or siRNA using Lipofectamine 2000 reagent (Invitrogen, Carlsbad, CA, USA) according to the manufacturer’s recommended protocol. The miRNA and siRNA included miR-937 inhibitor, miR-937 mimic, negative control (NC), pcDNA3.1-IL1B, and pcDNA3.1, which were all constructed from GenePharma Corporation (Shanghai, China). Transfection efficiency was measured 24 hours later after transfection.

### Total RNA isolation and RT-qPCR

Total RNA was extracted from cells using the EZNA RNA isolation kit (Onega Bio-Tek, Norcross, GA, USA), following the manufacturer’s recommended protocol.

For mRNA expression: 1 μg of isolated RNA was reverse-transcribed into cDNA by a PrimeScript RT reagent kit (Takara, Shiga, Japan). qRT-PCR was conducted using Power SYBR Green Master Mix (Applied Biosystems, Foster City, CA, USA) on the StepOnePlus RT-qPCR System. Relative expression was analyzed manually using the 2^–ΔΔCT^ method and normalized to the GAPDH. The sequences of primers used were as follows: IL1B, 5’-CCACAGACCTTCCAGGAGAATG-3' and 5’-GTGCAGTTCAGTGATCGTACAGG-3'; GAPDH, 5’-CCATGGGGAAGGTGAAGGTC-3' and 5’-GCAGGAGGCATTGCTGATGA-3'.

For miRNA expression: A TaqMan MicroRNA reverse transcription kit (Life Technologies, Carlsband, CA, USA) served to reverse transcribe total RNA to cDNA. qRT-PCR was carried out using the MiScript SYBR-Green PCR kit (Qiagen, Hilden, Germany) on the StepOnePlus RT-qPCR System. The expression of miR-937 was normalized to U6. The sequences of primers used were listed: miR-937, 5’-ATCCGCGCTCTGACTC-3' and 5’-GAACATGTCTGCGTATCTC-3'; U6, 5’-CTCGCTTCGGCAGCACAT-3' and 5'-TTTGCGTGTCATCCTTGCG-3'.

### Western blot

Total proteins were isolated from cells with a RIPA lysis buffer (Sigma-Aldrich, St. Louis, MO, USA) containing protease inhibitor on ice for minimizing degradation. Protein concentrations were quantified using Bio-Rad protein assay kit (Hercules, USA). Equal amounts of proteins, 20 μg in total, were separated on 10% SDS-PAGE. Then, samples were transferred onto PVDF membranes and blocked at room temperature with 5% skimmed milk powder for an hour. Next, primary antibodies (Beyotime) for IL1B, IL-6, IL-17, TNF-α, and GAPDH, were supplemented at a concentration of 1:1000 and incubated at 4^o^C for 24 hours. Upon rinsing three times with TBST, the membranes were incubated with the HRP-conjugated secondary antibodies for 1 hour at 37°C. Proteins were finally visualized using an ECL detection kit (Millipore, Billerica, MA, USA). ImageJ software (NIH, USA) served to quantify the gray values.

### Cell proliferation

The proliferation of HBE cells was assessed using cell counting kit 8 (CCK8, Beyotime, Jiangsu, China) reagent. After transfection or treatment for 24 hours, 1×103 cells per well were seeded in a 96 well plate and incubated for 24, 48, and 72 hours. At the indicated time points (0 , 24 , 48 , and 72 h), 10 μL of CCK8 reagent were added to each well and the plate was cultured in a carbon dioxide incubator for an hour at 37^o^C. The absorbance was recorded at 450 nm under a microplate reader (BioTek Instruments).

### Cell apoptosis

The apoptosis of HBE cells were assessed by double-staining with FITC-conjugated Annexin V and propidium iodide (PI). After treatment of 24 hours, cells were starved for 24 hours by serum-free culture medium when they grew to 50–60% confluence. Next, the cells were washed with cold PBS twice and centrifuged for 5 minutes at 1000 rpm. Subsequently, the cells were resuspended with 1× binding buffer and the concentration was adjusted to 1×10^6^ cells/mL. FITC-conjugated Annexin V (5 μL) and PI (5 μL) were added to 100 μL cell suspension, and then incubated in the dark for 5 minutes at room temperature. Immediately, the cell apoptosis was tested using flow cytometry (FACS Calibur, BD Biosciences, San Diego, CA) as instructed. The percentage of ‘Q2 plus Q4’ was identified as the cells apoptotic rate.

### Luciferase reporter assay

The binding sites between miR-937 and IL1B were predicted by TargetScan and confirmed by dual-luciferase reporter assay. The 3' UTR wild type or mutant type of IL1B with miR-937 binding sequences were cloned into pmir-GLO luciferase reporter vectors to construct IL1B-wt and -mut reporter vectors (pmir-GLO-IL1B-wt and pmir-GLO-IL1B-mut). HBE cells were plated into 24-well plates and co-transfected with 100 ng of these vectors and miR-937 mimic, miR-937 inhibitor or NC using Lipofectamine2000 (Invitrogen) as instructed. Following incubation for 24 hours, cells were collected and rinsed with PBS twice. Dual-luciferase reporter assay kit (Promega, Madison, WI, USA) was utilized to test the cells luciferase activity.

### Enzyme-Linked Immunosorbent Assays (ELISA)

The levels of IL-6, IL-17 and TNF-α in cell culture supernatants were detected by using ELISA kits (Beyotime Biotechnology, Shanghai, China) according to the manufacturer’s instructions. OD value at 450 nm (OD 450) was measured with a microplate reader (BioTek Instruments).

### Statistical analysis

All the experiments were done three times independently, and measurements are expressed as mean ± SD. Student’s t-test was utilized to compare the significant changes of two groups, while three or more groups were compared utilizing one-way ANOVA followed by *post hoc* test. A p<0.05 was identified as a significant change and indicated with an asterisk. All the analyses were carried out using GraphPad Prism 6.0 software (San Diegl, CA, USA).

## RESULTS

### Identification of candidate genes

**Figure 1:**
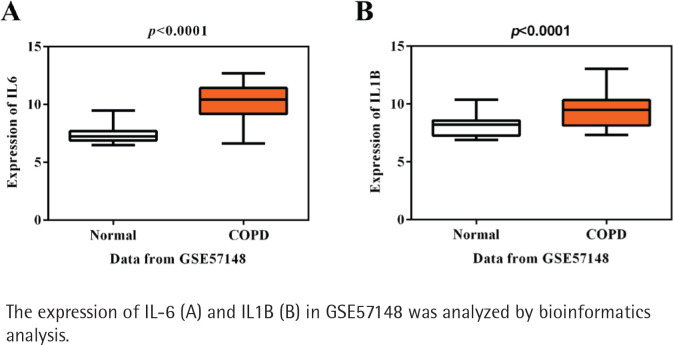
Higher expression of IL-6 and IL1B in COPD

Initially, using the mRNA expression profile GSE57148, a total of 459 genes were gained, containing 222 up-regulated and 237 downregulated DEGs in COPD patients compared with healthy controls. Next, KEGG pathway analysis of these DEGs presented IL-17 signaling, TNF signaling pathway, Salmonella infection, revealing their potential roles for the pathogenesis of COPD (Supplementary file Figure S1). The most enriched items of KEGG according to the corrected p-value and annotated genes are shown in Table 1. It is noted that IL-6 and IL1B are the most significant in these enriched pathways, suggesting the importance of these two inflammatory factors in COPD. As expected, the expressions of IL-6 and IL1B were both higher in COPD than healthy controls in accordance with GSE57148 (p<0.001, Figures 1 A-B). IL1B was chosen as our target due to its less reported studies in COPD in PubMed, compared with IL6, but the molecular mechanism of IL1B in COPD has not been fully elucidated.

**Table 1:** KEGG pathway analysis of target genes according to the GSE57148

*Pathway*	*Corrected p-value*	*Annotated genes*
Interleukin-17 signaling	1.68×10^-5^	IL6/S100A8/MMP1/IL1B
TNF signaling pathway	3.36×10^-5^	IL6/SOCS3/IL1B/JUNB
Salmonella infection	0.000330248	IL6/IL1B/DYNC1H1
Rheumatoid arthritis	0.000514587	IL6/MMP1/IL1B
Antifolate resistance	0.001301961	IL6/IL1B
Osteoclast differentiation	0.001305221	SOCS3/IL1B/JUNB
FoxO signaling pathway	0.00139555	IL6/SOD2/CDKN1A
Prion diseases	0.001659256	IL6/IL1B
African trypanosomiasis	0.001853516	IL6/IL1B

### miR-937 is the upstream regulatory miRNA of IL1B

**Figure 2:**
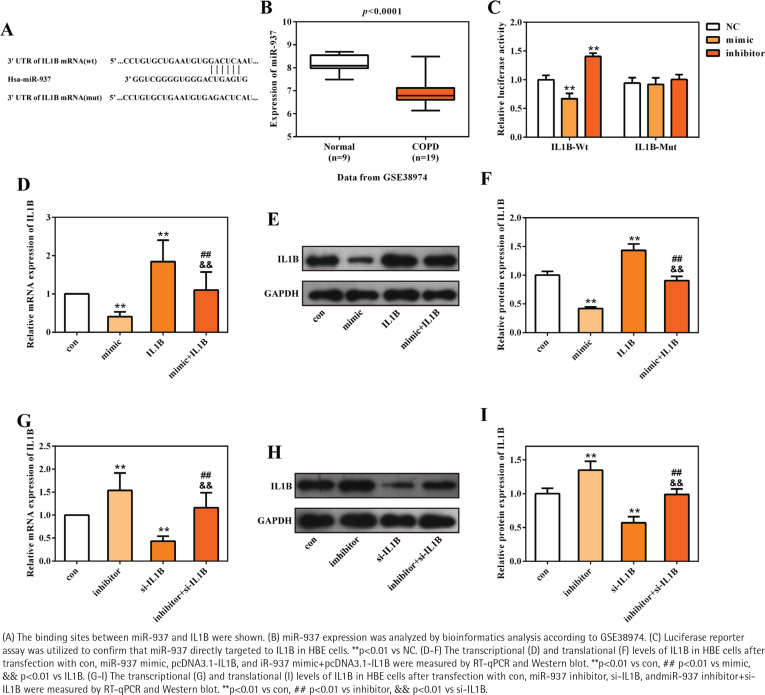
IL1B was a target of miR-937

A total of 17 differently expressed miRNAs in COPD containing 11 up-regulated and 6 downregulated miRNAs were obtained using the online miRNA expression profile GSE38974. The prediction website TargetScan (http://www.targetscan.org/) served to predict the upstream regulatory miRNAs of IL1B, and 226 miRNAs were found to target to the 3' UTR of IL1B. The intersection of this predicted results and 6 downregulated miRNAs demonstrated that miR-937 could be the potential upstream regulatory miRNA of IL1B. The sequences of hsa-miR-937 and the 3'UTR of IL1B are presented in Figure 2A, and Figure 2B shows the significant downregulation of miR-937 in COPD compared with healthy controls (p<0.001). Subsequently, the luciferase reporter vectors of IL1B-Wt and IL1B-Mut were constructed to verify the binding sites between miR-937 and IL1B via dual-luciferase reporter assay. The measurements showed that relative luciferase activity of the 3' UTR of IL1B wild-type was dramatically inhibited by miR-937 mimic whereas elevated by miR-937 inhibitor (p<0.01, Figure 2C). However, it was unchanged in miR-937 mimic, inhibitor and negative control groups, with co-transfection of 3' UTR of IL1B mutant-type (Figure 2C).

The transcriptional and translational levels of IL1B were determined in HBE cells through RT-qPCR and Western blot analyses. IL1B expression was negatively regulated by miR-937, as seen from the result that miR-937 mimic significantly downregulated while miR-937 inhibitor up-regulated the levels of IL1B (p<0.01, Figures 2 D-I). Importantly, the inhibitive/promotive effects of miR-937 mimic/inhibitor on the expression of IL1B were observed to be alleviated once IL1B-overexpression vectors or IL1B-knockdown siRNAs were introduced (p<0.01, Figures 2 D-I). Also, the mRNA and protein levels of IL1B were notably elevated after transfection with pcDNA3.1-IL1B whereas those were decreased after transfection with si-IL1B (p<0.01, Figures 2 D-I). All these detections indicated the directly, negatively regulatory function of miR-937 upon the 3' UTR of IL1B.

### miR-937 mediates the viability of HBE cells that inhibited by CSE

**Figure 3:**
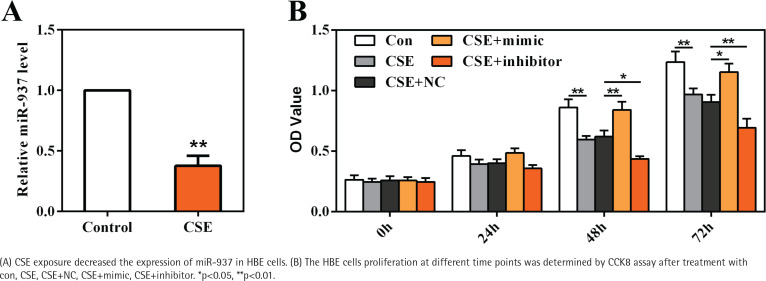
miR-937 was downregulated in CSE-induced HBE cells and affected cells proliferation

To gain an insight into the role of miR-937 in COPD, HBE cells were cultured and stimulated with 20 μg/mL CSE as an *in vitro* model. Results shown in Figure 3A reveal that miR-937 expression was markedly reduced by CSE (p<0.01), suggesting a potential role of miR-937 in CSE-treated HBE cells.

CCK8 assay was utilized to examine the role of miR-937 on cells proliferation. At 48 h and 72 h time points, the data showed that CSE-treated HBE cells displayed a notable decrease on the OD values (p<0.01, Figure 3B). Moreover, miR-937 mimic remarkably elevated cells proliferation, whereas miR-937 inhibitor notably reduced cells proliferation (p<0.05, Figure 3B). Together, these observations imply that miR-937 might participate in the regulation of CSE-induced HBE cells proliferation.

### The effects of miR-937 on the proliferation and apoptosis in CSE-treated HBE cells correlate with IL1B

**Figure 4:**
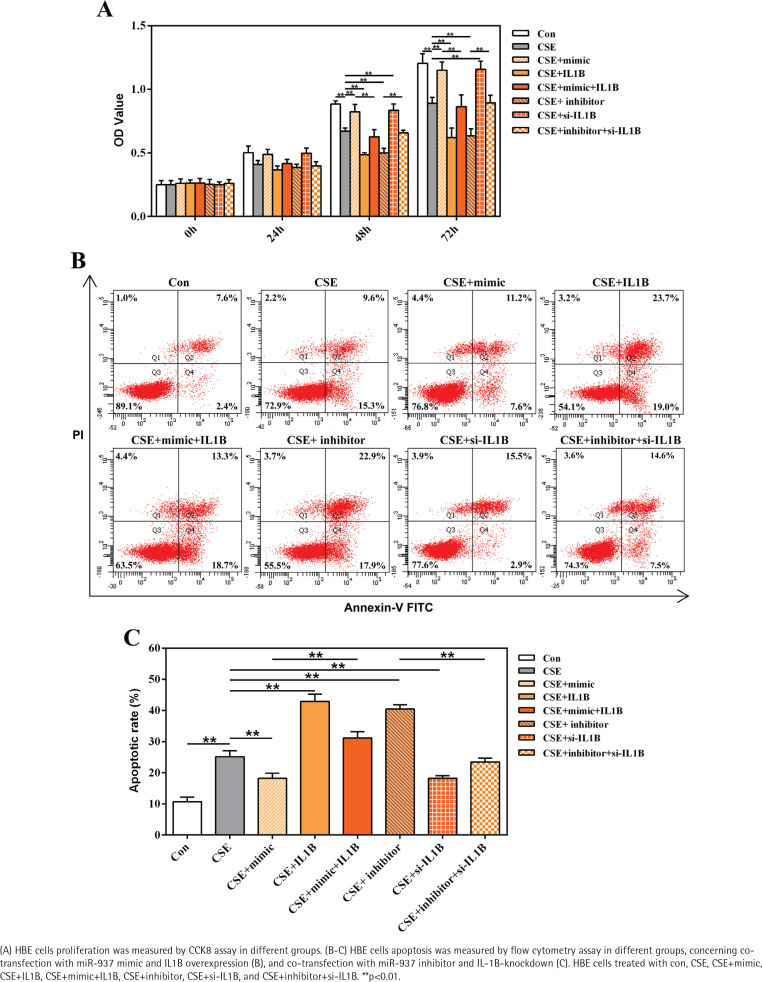
Effects of miR-937 on the CSE-induced HBEs proliferation and apoptosis associated with IL1B

The roles of miR-937/IL1B in the CSE-treated cells proliferation and apoptosis were measured by CCK8 and flow cytometry through gain-/loss-of-function approaches. Compared with controls, CSE notably inhibited HBE cells proliferation whereas enhanced cells apoptosis, which could be attenuated by introduction of miR-937 mimic or si-IL1B (p<0.01, Figure 4). However, after silencing miR-937 or overexpressing IL1B, cell proliferation was reduced while the apoptotic rate was raised compared to CSE controls (p<0.01, Figure 4). It is noteworthy that overexpression of IL1B weakened the effects of miR-937 mimic on enhancing proliferation and inhibiting apoptosis in CSE-treated HBEs (p<0.01, Figure 4). In addition, the impact of miR-937 inhibitor on CSE-treated HBEs activities could be abrogated by introduction of IL1B siRNA (p<0.01, Figure 4). All these findings suggest that miR-937 could help HBE cells against injury by CSE through targeting IL1B.

### The TNF-α/IL-17 signaling pathway is inhibited by miR-937/IL1B in CSE-induced HBE cells

**Figure 5:**
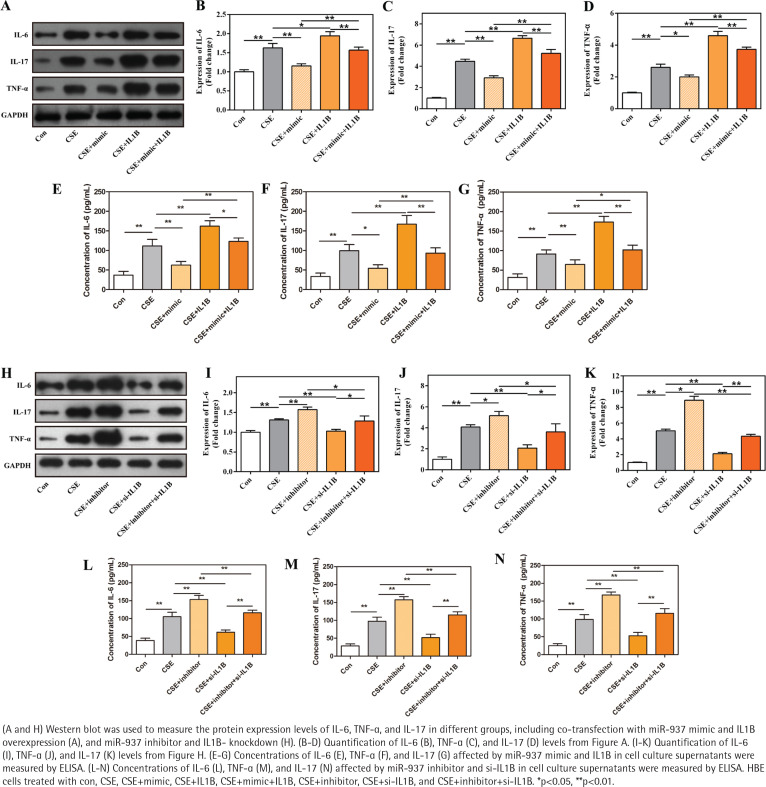
Effects of miR-937 on the expression of inflammatory factors in CSE-induced HBEs correlates with IL1B

Previous KEGG revealed that IL1B was enriched in TNF and IL-17 signaling pathways. To understand the involved molecular pathways, levels of IL-6, IL-17, and TNF-α, were detected by Western blot and ELISA analyses. Results shown in Figure 5 demonstrate that CSE activated the TNF-α/IL-17 signaling pathway, as measured, the expression of IL-6, IL-17, and TNF-α was up-regulated after CSE treatment in HBE cells compared with control (p<0.01). Furthermore, the results demonstrate that miR-937 mimic reduced the expression levels of these three inflammatory factors in HBE cells after CSE treatment (p<0.05, Figures 5 A-G). The suppressive effect of miR-937 mimic on the TNF-α/IL-17 signaling was reversed by overexpression of IL1B (p<0.01, Figures 5 A-G).

Analogously, when the miR-937 expression was suppressed under CSE conditions, the TNF-α/IL-17 pathway was activated, showing that IL-6, IL-17, and TNF-α were elevated importantly, these effects can be weakened by the specific si-IL1B (p<0.05, Figures 5 H-N). Besides, overexpression of IL1B in HBEs after CSE treatment, the expressions of these three markers were significantly increased (p<0.05, Figures 5 A-G), whereas siRNA-mediated IL1B silencing resulted in the declines of these expressions (p<0.01, Figures 5 H-N). Hence, the results suggest that miR-937 might be involved in the progression of CSE-induced HBE cells by targeting IL1B via inactivating the TNF-α/IL-17 signaling pathway.

## DISCUSSION

COPD, characterized by chronic and progressive dyspnea, is a threat to public health^22^. Increasing evidence has shown that genetic risk and environmental factors contribute to the pathogenesis of this disease^23^. Elucidation of its potential molecular mechanism would provide more valuable reference information for clinical treatment. The purpose of this study was to characterize the role of miR-937 and the molecular mechanism in the pathogenesis of HBEs stimulated by CSE, providing a novel mechanistic explanation for the role of miR-937 in the progression of COPD.

Recently, the studies of therapeutic targets and mechanism for COPD at molecular levels have aroused widespread interest. As we know, numerous miRNAs have been disclosed to be involved in the pathogenesis of COPD. Since COPD is a multifactorial disease with chronic inflammation as a dominant character^24^, exploring the relationship between miRNAs and inflammatory factors may help us understand the pathogenesis of COPD. Previous study revealed that miR-937 was remarkably downregulated in COPD patients compared with non-smokers via searching public databases^18^. However, a detailed molecular understanding of miR-937 function in the cells progression is still lacking. Here, according to a previous report, miR-937 was also found to be downregulated in COPD patients. Furthermore, inflammatory factor IL1B was verified as a target of and negatively regulated by miR-937. Since smoking is a key irritant in the occurrence and development of COPD^25^, CSE was utilized to simulate smoking in HBE cells to construct *in vitro* model. Besides, numerous studies have revealed that direct exposure to CSE influences miRNAs expressions in COPD, such as miR-195^24^, miR-212^26^, and miR-145^27^. According to these concepts, this study also indicated that direct exposure to CSE downregulated the miR-937 expression, and reduced the HBE cells proliferation whereas initiated apoptosis. The more important finding is that miR-937 has anti-apoptosis and pro-proliferation effects in the progression of HBE cells by targeting IL1B, hinting that upregulation of miR-937 may exhibit a protective effect on the CSE-induced HBEs.

Generally, various inflammatory cytokines, such as IL-6, IL-1B, IL-17 and TNF-α can lead to impairment of clearance ability of apoptotic cells^28,29^, suggesting that abnormal expressions of these inflammatory cytokines may play a crucial role in the development of COPD. In the present study, IL1B was identified to be linked with the TNF and IL-17 signaling pathway or pathways, which were the most enrichment significant pathways. Yi et al.^11^ has identified that IL1B is highly related to COPD disease trait, and it is highly expressed in COPD small airway epithelial cells. Zou et al.^30^ concluded that high serum levels of IL1B and IL-17 may be implicated to persistent neutrophilic airway inflammation. Here, elevated expression of IL1B was also found to reduce cells proliferation and elevate cells apoptosis after CSE treatment. Besides, the effects of miR-937 on the proliferation and apoptosis in CSE-treated HBE cells were rescued by restoration of IL1B. Our results further support the possibility that drugs reducing IL1B levels may have implications for clinical management of COPD^31^.

IL1B is an important driver in COPD, mainly involved in the mechanism where, in the CS-induced pulmonary inflammatory model, IL1B mediated the increasing number of dendritic cells and T-lymphocytes in bronchoalveolar gavage fluid and lung tissue, and these two types of cells could promote the release of IL-6 and IL-17, increasing the recruitment of neutrophils into the airway^32,33^. Lung tissue in COPD is mainly manifested in inflammatory patterns, with an increase in a variety of inflammatory cytokines, including TNF-α, which is one of the most studied cytokines in COPD research^34^. Moreover, TNF-α could induce the release of other proinflammatory factors^35^. Due to the correlation between IL1B and IL-17/TNF pathway, analyzed by bioinformatics analysis, it was examined whether miR-937/IL1B was implicated in the activation or inactivation of the inflammatory pathways. Western blot and ELISA showed that miR-937/IL1B could affect the protein expressions of IL-6, IL-17, and TNF-α in the CSE-damaged HBEs, associated with the TNF-α/IL-17 signaling pathway. These make sense to understand the molecular mechanisms of miR-937 more specifically, suggesting that miR-937 may be a potential therapeutic target to improve COPD. In line with the above reports, our detections showed that miR-937 promoted the proliferation and inhibited the apoptosis of HBEs caused by CSE, which may function through the TNF-α/IL-17 signaling pathway by targeting IL1B.

## CONCLUSIONS

The present study revealed that miR-937 could reduce HBE cells injury after CSE treatment via suppressing the TNF-α/IL-17 signaling pathway by targeting IL1B, indicating that miR-937 may have the potential to improve the therapeutic approaches of COPD. Our results provide reference information with respect to understanding the pathogenesis of COPD.

## Data Availability

The data supporting this research is available from the author on reasonable request.
