# Supplementary material for: miR-937 serves as an inflammatory inhibitor in cigarette smoke extract-induced human bronchial epithelial cells by targeting IL1B and regulating TNF-α/IL-17 signaling pathway
Source: Tob Induc Dis. 2021 Jun 25;19:55. doi: 10.18332/tid/138227 (PMC8231861; doi:10.18332/tid/138227)
Supplement: Supplementary file 1 [file TID-19-55-s1.pdf]

Figure S1 KEGG pathway enrichment analysis of overlapping DEGs

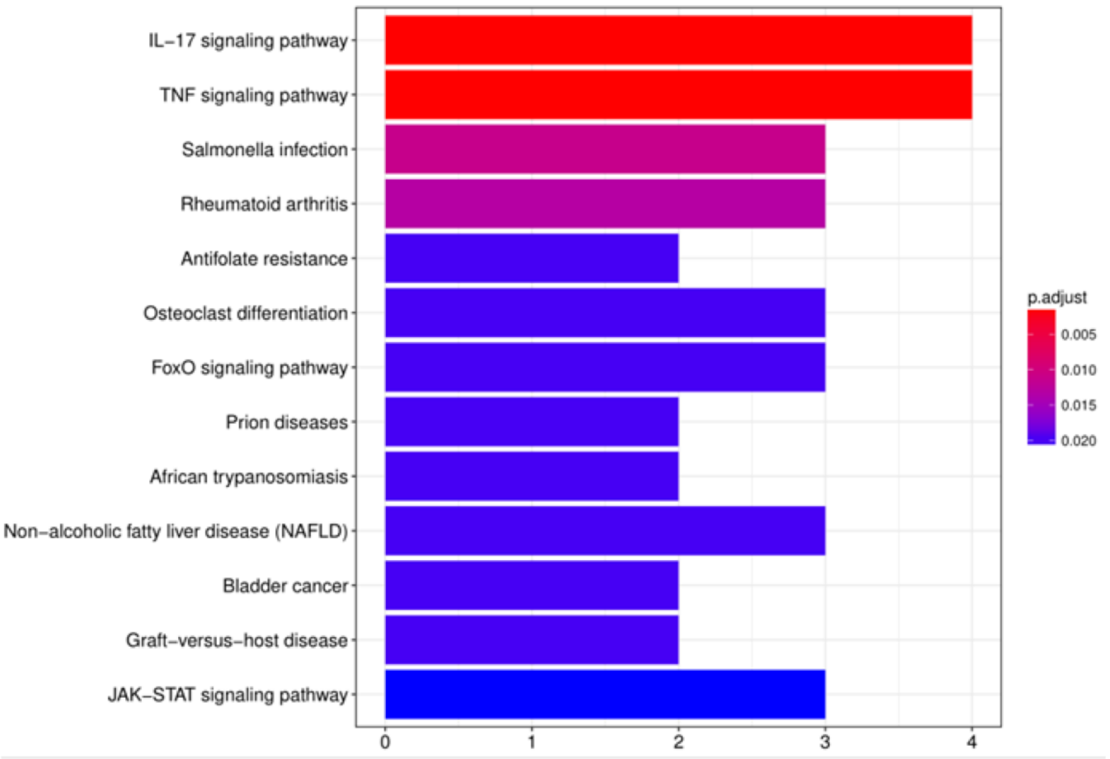

X axis represents number of genes enriched in KEGG pathways, y axis shows the names of pathways. Colors of columns represents the significance by adj.*p*-value.

© 2021 Liu T.
